# Association between Follicular Fluid Leptin and Serum Insulin Levels in Nonoverweight Women with Polycystic Ovary Syndrome

**DOI:** 10.1155/2014/980429

**Published:** 2014-05-04

**Authors:** G. Garruti, R. de Palo, M. T. Rotelli, S. Nocera, I. Totaro, C. Nardelli, M. A. Panzarino, M. Vacca, L. E. Selvaggi, F. Giorgino

**Affiliations:** ^1^Section of Internal Medicine, Endocrinology, Andrology and Metabolic Diseases, Department of Emergency and Organ Transplantation, University of Bari Aldo Moro, Piazza G. Cesare 11, 70124 Bari, Italy; ^2^Centre of Pathophysiology of Human Reproduction and Gametes Cryopreservation, Department of Gynaecology, Obstetrics and Neonatology, Gynaecology and Obstetrics Unit A, University of Bari Aldo Moro, Piazza G. Cesare 11, 70124 Bari, Italy

## Abstract

*Aims*. We evaluated the links between leptin and visfatin levels and fertilization rates in nonoverweight (NOW) women with PCOS (NOW-PCOS) from Apulia undergoing in vitro fertilization/embryo transfer (IVF). *Materials and Methodology*. We recruited 16 NOW women with PCOS (NOW-PCOS) and 10 normally ovulating NOW women (control-NOW). All women underwent IVF. Androgens, 17-**β**-estradiol (17**β**-E_2_), and insulin levels were measured in plasma and/or serum and leptin and visfatin levels were assayed in both serum and follicular fluid (FF-leptin, FF-visfatin). *Results*. In NOW-PCOS, both serum and FF-leptin were significantly lower than in control-NOW. In NOW-PCOS, significant correlations were found between BMI and serum leptin and insulinemia and FF-leptin. By contrast, in control-NOW, FF-leptin levels were not correlated with insulinemia. Serum visfatin levels were not significantly different in NOW-PCOS and control-NOW, but FF-visfatin levels were 1.6-fold higher, although not significantly, in NOW-PCOS than in control-NOW. *Conclusions*. Both serum leptin levels and FF-leptin are BMI- and insulin-related in Southern Italian NOW-PCOS from Apulia. In line with other reports showing that FF-leptin levels are predictive of fertilization rates, lower than normal FF-leptin levels in NOW-PCOS may explain their lower fertilization rate and this may be related to the level of insulin and/or insulin resistance.

## 1. Background

Polycystic ovary syndrome (PCOS) is commonly defined, according to the Amsterdam ESHRE/ASRM PCOS Consensus Workshop Group [1], as a disorder including anovulation, menstrual disturbances, and hyperandrogenism and is often associated with clinical features of the metabolic syndrome with an increased risk of type 2 diabetes. PCOS is the major cause of infertility in women in the fertile age. Studies on different ethnic groups support a familial and genetic predisposition to PCOS, and oligogenic/polygenic mechanisms might be involved in its etiology [2, 3]. Both hyperinsulinemia and/or insulin resistance and hyperandrogenism play an important role in its pathogenesis [4]. In addition, obesity is often, but not always, a fellow traveller of PCOS.

In* ob/ob* mice, displaying a phenotype similar to that of human obesity, a point mutation of the* ob* gene encoding for leptin, accounts for hyperphagia, morbid obesity, and sterility [5, 6]. Interestingly, humans that are homozygous for a point mutation of the* ob* gene also display a phenotype whose main features are morbid obesity and sterility [7, 8]. Impaired function and/or reduced levels of leptin might thus cause infertility in women with PCOS. Consistent with this concept, circulating leptin levels and leptin mRNA expression in adipocytes are reduced in women with PCOS [9, 10].

Several studies have investigated leptin levels in overweight and/or obese women with PCOS, clearly demonstrating that they appear to be correlated with BMI [11]. However, granulosa cells are able to synthesize and store leptin from newborn until adult life in a manner that is independent of BMI [12]. Relevant to this concept, few studies have investigated leptin levels in normal-weight women with PCOS in specific ethnic groups in the absence of excess BMI. In an Indian study, circulating leptin levels were found to be 5-fold higher in normal-weight women with PCOS as compared with controls [13]. In a Chinese study, PCOS women were characterized by higher leptin levels both in serum and follicular fluid than controls, although the BMI of the PCOS women included in this study ranged from normal-weight to overweight and obesity [14]. The above-mentioned data are not in line with other results in Caucasian women demonstrating that the concentration of leptin in the follicular fluid (FF-leptin) seems to be predictive of the fertilization rate [15]. Fertilization rate is usually lower in PCOS women as compared with normally ovulating women. The paper by De Placido et al. [15] is the only one to find a direct correlation between the concentration of leptin in the follicular fluid (FF-leptin) and the fertilization rate. Interestingly the population studied by De Placido et al. was coming from the south of Italy and certainly showed different lifestyle and diet as compared with Indians and Chinese women.

In other recent studies, elevated circulating levels of leptin and/or increased leptin/adiponectin ratios were reported in overweight PCOS women, but no information on leptin levels in lean women with PCOS was provided [16, 17].

Visfatin acts as a cytokine but also as an enzyme and is involved in metabolic and immunological diseases. Visfatin mRNA was recently detected in human follicles, and visfatin administration was shown to improve ovarian response in conditions of superovulation in animal models [18, 19].

In this study, we have measured circulating leptin and visfatin levels and FF-leptin and FF-visfatin in a selected group of Caucasian Southern Italian nonoverweight women with PCOS (NOW-PCOS) undergoing in vitro fertilization/embryo transfer (IVF) in order to better understand the interrelationships, if any, between both cytokines and fertilization rate.

Some other papers analyzed leptin levels in both serum and follicular fluid in women having PCOS; however, very few papers included only Caucasian nonoverweight women (BMI ≤ 24,9 Kg/m^2^) with PCOS as we did. Our pilot study is also new in the fact that all women studied were coming from Apulia, a region from the south of Italy which is very well known for Mediterranean diet.

## 2. Materials and Methods

In this observational pilot study, we selected 16 consecutive NOW-PCOS and 10 consecutive age- and BMI-matched normal-ovulating nonhirsute premenopausal infertile Caucasian women (control-NOW) requesting a procedure of assisted reproduction at the Centre of Pathophysiology of Human Reproduction and Gametes Cryopreservation of the University of Bari. All women were from Apulia, in Southern Italy, and did not show any sign of metabolic syndrome. In the control-NOW group, infertility was due either to male infertility or tubal factors. The diagnosis of PCOS was based on the criteria indicated in the Amsterdam ESHRE/ASRM-Sponsored 3rd PCOS Consensus Workshop Group [1, 20]. All women with PCOS had hirsutism, oligomenorrhea, and clear diagnosis of PCOS by ultrasounds. Both NOW-PCOS and control-NOW were euthyroid and were undergoing IVF for the first time. All women underwent IVF according to a previously reported protocol [21]. Infertile women with a diagnosis of diabetes mellitus were excluded from the study to rule out potential confounding effects of hyperglycemia on leptin and fertilization rates. Indeed, all subjects had fasting glucose levels <126 mg/dL (7 mmol/L) and underwent an oral glucose tolerance test according to the American Diabetes Association guidelines [22], displaying plasma glucose levels <140 mg/dL (7.8 mmol/L) after 2 h. All women were in the premenopausal stage, as confirmed by circulating FSH and LH levels measured between day 3 and day 5 of the menstrual cycle. All women underwent IVF according to a previously reported protocol [21]. Briefly, daily GnRH-a administration (Decapeptyl 0.1 mg/mL-Ipsen S.p.A., s.c.) was started during the middle-luteal phase of the cycle before ovarian hyperstimulation until the day of human chorionic gonadotropin (hCG) administration. Ovarian hyperstimulation was initiated once the serum estradiol (E_2_) level was <30 pg/mL with 150–225 IU of rFSH (alpha follitropin-Gonal-F, Merck Serono S.p.A.). Dosing was individualized depending on the patient's response. When at least 3 follicles of 18 mm in diameter and a serum E_2_ level of >200 pg/mL per follicle were noted, the patient was given 250 *μ*g of choriongonadotropin alpha hormone (Ovitrelle, Merck Serono S.p.A.). Monitoring was accomplished with serial vaginal ultrasound examinations and serum E_2_ level determinations. Transvaginal oocyte retrieval was performed 35 hours after hCG injection. After retrieval, cumulus cells (COCs) were removed with a 30-second exposure to HEPES-buffed medium containing 40 IU/mL hyaluronidase (SAGE, USA), and denuded oocytes were then assessed for nuclear status: those that were observed to have released the first polar body were considered mature (MII) and used for ICSI procedure [23].

Fertilization was assessed 18 hours after ICSI. Normal fertilization was confirmed when 2 clearly distinct pronuclei were present. Fertilization rate was defined as the percentage of pronuleate oocytes to the number of oocytes injected. Embryo quality was evaluated under inverted microscope on the second day of development according to Veeck [24]. A maximum of three embryos were transferred on day 2 or 3 of embryo culture under ultrasound guidance. For luteal phase support all patients received a daily dose of 400 mg of vaginal micronized progesterone (Progeffik, Effik Italia). Serum *β*-hCG concentration was measured 14 days after oocyte retrieval to verify pregnancy. Clinical pregnancy was judged by observation of the gestational sac on vaginal ultrasonography after 6-7 weeks of gestation.

Follicular fluid (FF) samples were obtained during IVF treatment at the time of oocyte aspiration. Bloodless aspirates from different follicles containing mature oocytes of one patient were pooled and centrifuged (1,200 rpm for 5 min) (for 10 min at 2,000 g). The supernatants were divided in aliquots and stored in sterile tubes and stored at −80°C.”

Blood samples for metabolic and hormonal evaluations were collected before the IVF stimulation protocol. Total cholesterol, HDL-cholesterol, LDL-cholesterol, and triglycerides circulating levels were measured with specific Dimension clinical chemistry systems (Siemens Healthcare Diagnostics Ltd.). Androgens (free and total testosterone, Δ4-androstenedione, SHBG, DHT, DHEA, and DHEAS) were measured between day 3 and day 5 of the menstrual cycle. 17*β*-E_2_ and insulin levels were measured in plasma and/or serum and leptin and visfatin levels in both serum and follicular fluid (FF) (DIAsource Leptin-EASIA Kit, Visfatin-EASIA Kit).

The homeostasis model assessment of insulin resistance (HOMA-IR) index was calculated, as previously reported [25, 26].

The fertilization rates were calculated and correlated with the anthropometric and hormonal parameters.

All results are expressed as mean ± SEM. Comparison of continuous variables among groups was performed with paired Student's *t*-test. All statistical calculations were performed with NCSS2004 software (Kaysville, UT, USA). Statistical tests were conducted as an alpha level of 0.05 [27, 28]. A stepwise regression analysis was performed to identify significant links between anthropometric and metabolic variables and between the above mentioned variables and fertilization rates.

## 3. Results

In this study, we have investigated NOW-PCOS, who requested a procedure of assisted reproduction, with no signs of metabolic syndrome. Both NOW-PCOS and control-NOW had total cholesterol, HDL-cholesterol, LDL-cholesterol, and triglycerides levels as well as arterial blood pressure levels in the normal range and showed a normal glucose tolerance (data not shown).

**Figure 1:**
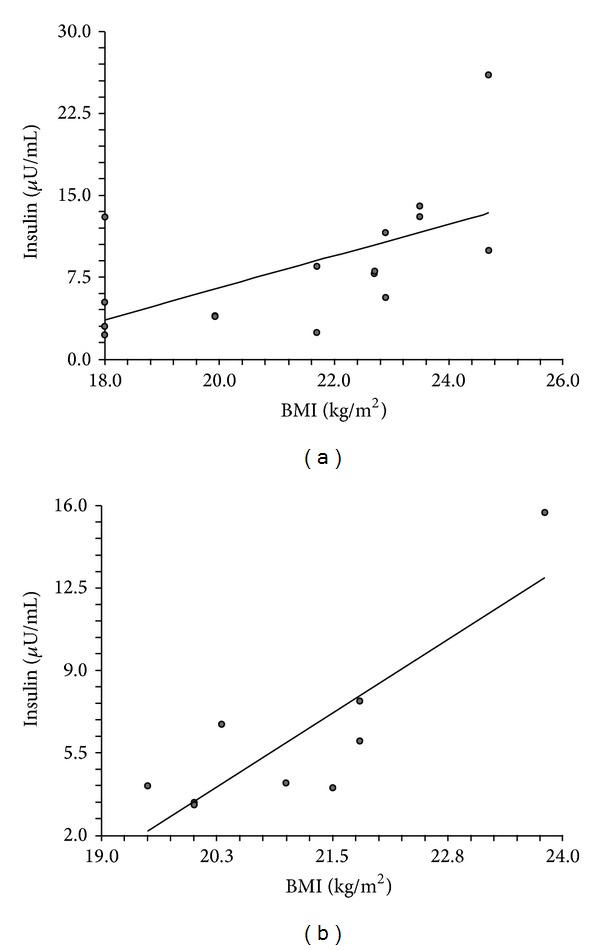
Correlation between circulating insulin levels and BMI in NOW-PCOS and control-NOW. (a) Positive linear correlation between circulating insulin levels and BMI in NOW-PCOS. *R* ^2^ = 0.3385; *r* = 0.5818; *p* = 0.0181. (b) Positive linear correlation between circulating insulin levels and BMI in control NOW. *R* ^2^ = 0.7169; *r* = 0.8467; *p* = 0.0040.

NOW-PCOS and control-NOW had comparable fasting glucose and insulin levels, and HOMA-IR was also not significantly different in the two groups (Table 1). As far as circulating insulin levels are concerned they were significantly correlated with BMI in both NOW-PCOS and control-NOW (Figures 1(a) and 1(b)).

**Figure 2:**
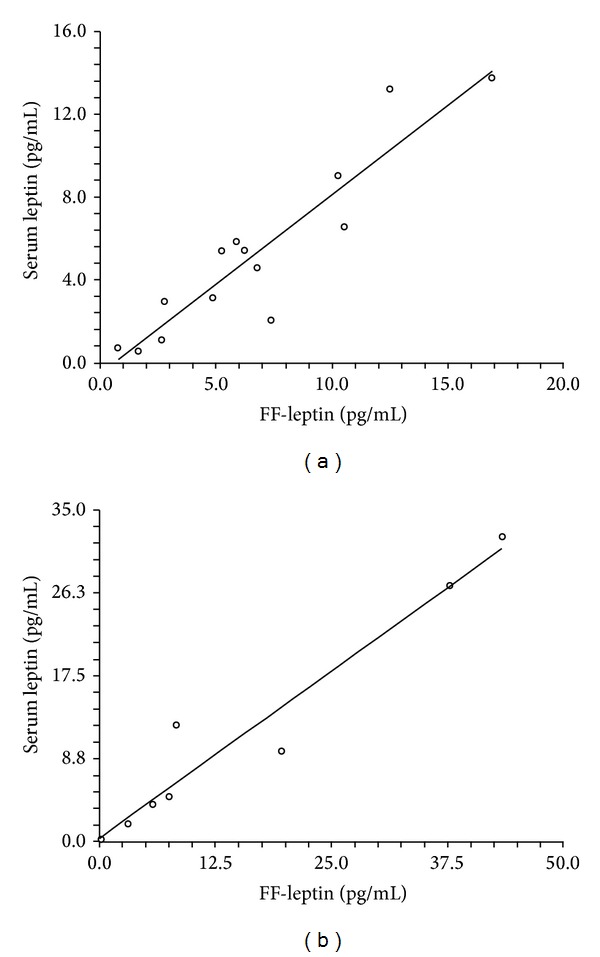
Correlation between leptin levels in the follicular fluid and circulating leptin levels in NOW-PCOS and control-NOW. (a) Positive linear correlation between leptin levels in the follicular fluid and circulating leptin levels in NOW-PCOS. *R* ^2^ = 0.8517; *r* = 0.9229; *p* = 0.00001. (b) Positive linear correlation between leptin levels in the follicular fluid and circulating leptin levels in control-NOW. *R* ^2^ = 0.9376; *r* = 0.9683; *p* = 0.0001.

**Figure 3:**
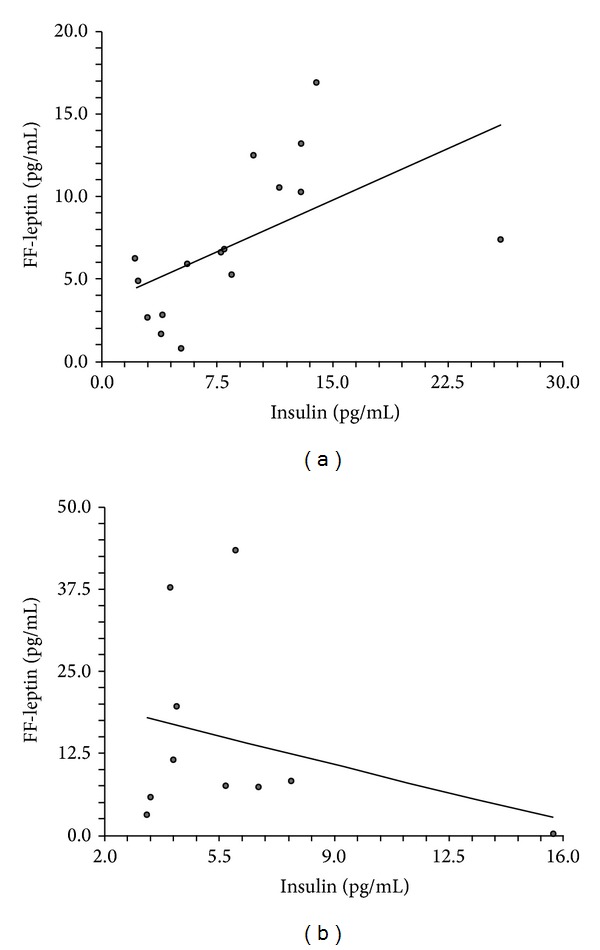
Correlation between leptin levels in the follicular fluid and circulating insulin levels in NOW-PCOS and control-NOW. (a) Positive linear correlation between leptin levels in the follicular fluid (FF) and circulating insulin levels in NOW-PCOS. *R* ^2^ = 0.3173; *r* = 0.5633; *p* = 0.0231. (b) Absence of correlation between leptin levels in FF and circulating insulin levels in control-NOW. *R* ^2^ = 0.0919; *r* = −0.3032; *p* = 0.3944.

In both NOW-PCOS and control-NOW, serum leptin levels positively correlated with FF-leptin levels (Figures 2(a) and 2(b)). A positive linear correlation between FF-leptin and circulating insulin levels was found in NOW-PCOS but not in control-NOW (Figures 3(a) and 3(b)).

In our cohort of NOW-PCOS, a significant positive correlation was found between BMI and serum leptin (*R* ^2^ = 0,3838; *r* : 0.6195; *p* = 0.0181) and a positive, although nonsignificant, correlation was also observed between BMI and FF-leptin (*R* ^2^ = 0, 2379, *p* = 0, `0553).

As far as visfatin is concerned, serum visfatin levels were not different in NOW-PCOS and control-NOW, but FF-visfatin levels were 1.6-fold higher, even though not significantly, in NOW-PCOS as compared with control-NOW (*p* = 0.075, Table 1). No correlation was found between FF-visfatin and circulating insulin, HOMA-IR, and FF-leptin in either NOW-PCOS or control-NOW.

Basal nonstimulated FSH levels were not significantly different between the two groups, but basal nonstimulated LH levels were significantly higher in NOW-PCOS than control-NOW (*p* = 0.001, Table 1), and basal 17*β*-E_2_ levels were 2.9-fold lower in NOW-PCOS as compared to control-NOW, even though this difference was not statistically significant (*p* = 0.07) (Table 1). In regard to the IVF stimulation procedure, the number of total oocytes was significantly higher (*p* = 0.001) but in-vitro-matured metaphase II oocytes were lower, although not significantly (*p* = 0.07), in NOW-PCOS than in control-NOW (Table 2). Even if fertilization rate has been known to be lower in PCOS women as compared with normally ovulating women, due to IVF, the fertilization rate resulted to be not different in the two groups. At the end of the IVF protocol, estrogen levels were threefold higher in NOW-PCOS than in control-NOW (*p* = 0.001).

## 4. Discussion

PCOS is highly prevalent (about 4–7%) among women in the reproductive age [25, 26] and recognizes in the impaired hypothalamus-pituitary-ovarian axis dysfunctions and hyperinsulinemia major causes driving the infertile phenotype. Nonetheless, overweight and obesity are often, but not always, associated with PCOS, and adipokines, including leptin and visfatin, might also be involved in the pathogenesis of this disease [18–20, 29–34].

Some previous reports suggest that adipokines might be involved in the pathogenesis of PCOS [18, 31]. Since, in both animal and human models of leptin deficiency, infertility is present together with the obesity phenotype [5–8], it is to be expected that altered levels and/or function of leptin might be involved in causing infertility in overweight women with PCOS too. Leptin levels usually correlate with body fat mass, and since PCOS is often associated with overweight, the latter might represent a confounding factor in the effort to understand the role of leptin in the pathogenesis of PCOS. In this study, we investigated strictly nonoverweight women requesting a procedure of assisted reproduction and excluded both overweight and obese women to rule out potential confounding effects of overweight and obesity on leptin and/or visfatin levels and fertilization rates. We studied a small group of homogeneous Italian-Caucasian women from Apulia. We found significantly lower serum and FF-leptin levels in NOW-PCOS compared to BMI-matched infertile women and a positive correlation between insulinemia and FF-leptin levels in NOW-PCOS only.

In studies performed in non-Caucasian ethnic groups (Chinese, Indian), higher levels of leptin in both serum and FF were reported in PCOS women than in controls; however, in these studies the experimental groups included normal-weight, overweight, and obese PCOS women (range 19.81–30.62 Kg/m^2^) [13, 14]. In another recent study on Caucasian women with PCOS, elevated circulating levels of leptin and/or increased leptin/adiponectin ratios were found in overweight PCOS women, but leptin levels in lean women with PCOS were not investigated [16].

Nonetheless, results on interethnic differences in body fat distribution and phenotype make it reasonable to find different adipokine levels in different ethnic groups, particularly when different cutoff values for waist circumferences have to be considered [17].

It is interesting to note that, in another study involving Caucasian PCOS women from Southern Italy, FF-leptin concentrations were found to be predictive of the fertilization rates [15]. In PCOS women, the fertilization rate is known to be lower than in normally ovulating women, and therefore it may seem reasonable to find reduced FF-leptin levels in these subjects.

In our study, both NOW-PCOS and control-NOW had normal glucose tolerance. Fasting insulin levels and HOMA-IR index were not significantly different in the two groups. However, circulating insulin levels correlated with FF-leptin in NOW-PCOS only, even if in both NOW-PCOS and control-NOW they correlated with BMI.

It has been already demonstrated that hyperleptinemia and/or high FF-leptin may result in impaired steroidogenesis, reproductive function, and fertility [15, 33, 34]. However, it is also true that follicle maturation occurs in the presence of physiologic levels of leptin through the activation of the STAT signal transduction pathway [13, 15]. The possibility exists that too low levels of FF-leptin decrease fertility in NOW-PCOS women as well as too high FF-leptin levels might favor infertility in overweight/obese PCOS women. In overweight PCOS women, insulin resistance is certainly associated with increased steroidogenesis, even if the adipose cell lineage is not intrinsically insulin-resistant [35, 36].

As far as visfatin is concerned, it belongs to the family of cytokines and has been implicated in several metabolic conditions, such as central obesity, metabolic syndrome, and type 2 diabetes. In an animal model (mouse), administration of visfatin during superovulation improves developmental competency of oocytes and fertility potential, despite the age. These and other data support a role for visfatin in reproduction [18, 19]. In our experimental cohort, serum visfatin levels were not different in NOW-PCOS and control-NOW, but FF-visfatin levels were 1.6-fold higher, even though not significantly, in NOW-PCOS as compared with control-NOW.

In regard to the IVF stimulation procedure, even if fertilization rate has been known to be lower in PCOS women as compared with normally ovulating women, due to IVF, the fertilization rate resulted to be not different in the two groups of women analysed in our study. Since during the IVF protocol, NOW-PCOS and control-NOW followed a lifestyle change program including a Mediterranean low-glycemic index isocaloric diet and daily moderate exercise, according to standard care protocols, we can argue that this lifestyle change might have resulted in improved fertilization rates in women with PCOS, even if estrogen levels were still higher in NOW-PCOS than in control-NOW.

A possible take-home message to get from our pilot study is that NOW-PCOS might represent a completely different phenotype as compared with those found in overweight or obese women with PCOS. FF-leptin levels lower and FF-visfatin levels higher than those found in control-NOW might be markers of this different phenotype and low fertilization rate.

Considering the correlation between insulin circulating levels and FF-leptin in NOW-PCOS, future studies should more closely focus on direct measurements of insulin sensitivity and insulin secretion in NOW-PCOS to better elucidate the relationship between changes in insulin secretion and/or action and changes in cytokines relevant to the fertility status in this population.

## Figures and Tables

**Table 1:** Anthropometric and metabolic characteristics and hormone levels in the experimental subjects.

	NOW-PCOS	Control-NOW	*p* value
Age (years)	32.09 ± 3.60	34.35 ± 3.13	**0.04**
BMI (Kg/m^2^)	22.55 ± 2.61	22.55 ± 2.55	0.99
Insulinemia (*μ*U/mL)	10.30 ± 7.44	9.39 ± 9.27	0.73
HOMA-IR	2.57 ± 2.06	2.37 ± 2.72	0.76
FSH (mlU/mL)	5.71 ± 2.02	6.96 ± 2.34	0.07
LH (mlU/mL)	8.12 ± 4	4.77 ± 1.61	**0.001**
17*β*-E_2 _(basal) (pg/mL)	32.6 ± 13.40	95.56 ± 131.63	0.07
FF-leptin (ng/mL)	7.12 ± 1.12	14.44 ± 4.67	**0.037**
Serum leptin (ng/mL)	5.31 ± 1.13	11.49 ± 4.21	**0.045**
FF-visfatin (ng/mL)	4.55 ± 0.59	2.88 ± 0.88	0.075
Serum visfatin (ng/mL)	8.66 ± 0.81	7.24 ± 1.48	0.74

NOW: nonoverweight; control-NOW: controls; BMI: body mass index; E_2_: estradiol; FF: follicular fluid; FSH: follicle stimulating hormone; LH: luteinizing hormone. Data are expressed as mean ± SEM. Differences between groups were considered significant for *p* ≤ 0.05.

**Table 2:** Outcomes at the stimulation end in the experimental women.

	NOW-PCOS	Control-NOW	*p* value
Follicles <18 mm	8.14 ± 3.07	2.58 ± 2.48	2.11
Follicles >18 mm	6.24 ± 2.02	6.05 ± 2.55	0.8
17*β*-E_2_ (pg/mL)	3184.26 ± 1477.09	1689.67 ± 1075.79	**0.001**
Total oocytes	9.33 ± 3.28	5.95 ± 2.86	**0.001**
M II	3.76 ± 1.37	4.89 ± 2.26	**0.07**
M II/total oocytes (%)	45.38 ± 22.78	81.23 ± 23.73	2.12
Fertilization rate (%)	60.16 ± 35.22	62.82 ± 33.77	0.81
Embryos (I°)	1.74 ± 1.52	2.05 ± 1.47	0.52
Pregnancy rate (%)	30	35	

NOW: nonoverweight; control-NOW: controls; BMI: body mass index; E_2_: estradiol; M II: in-vitro-matured metaphase II oocytes. Data are expressed as mean ± SEM. Differences between groups were considered significant for *p* < 0.05.

## References

[1] Amsterdam ESHRE/ASRM-Sponsored 3rd PCOS Consensus Workshop Group (2012). Consensus on women's health aspects of polycystic ovary syndrome (PCOS). *Human Reproduction*.

[2] Legro RS, Driscoll D, Strauss JF, Fox J, Dunaif A (1998). Evidence for a genetic basis for hyperandrogenemia in polycystic ovary syndrome. *Proceedings of the National Academy of Sciences of the United States of America*.

[3] Ewens KG, Jones MR, Ankener W (2011). FTO and MC4R gene variants are associated with obesity in polycystic ovary syndrome. *PLoS ONE*.

[4] Dunaif A, Wu X, Lee A, Diamanti-Kandarakis E (2001). Defects in insulin receptor signaling in vivo in the polycystic ovary syndrome (PCOS). *American Journal of Physiology: Endocrinology and Metabolism*.

[5] Zhang Y, Proenca R, Maffei M, Barone M, Leopold L, Friedman JM (1994). Positional cloning of the mouse obese gene and its human homologue. *Nature*.

[6] Frϋhbeck G (2006). Intracellular signalling pathways activated by leptin. *Biochemical Journal*.

[7] Montague CT, Farooqi IS, Whitehead JP (1997). Congenital leptin deficiency is associated with severe early-onset obesity in humans. *Nature*.

[8] Strobel A, Issad T, Camoin L, Ozata M, Strosberg AD (1998). A leptin missense mutation associated with hypogonadism and morbid obesity. *Nature Genetics*.

[9] Mantzoros CS, Dunaif A, Flier JS (1997). Leptin concentrations in the polycystic ovary syndrome. *Journal of Clinical Endocrinology and Metabolism*.

[10] Lecke SB, Mattei F, Morsch DM, Spritzer PM (2011). Abdominal subcutaneous fat gene expression and circulating levels of leptin and adiponectin in polycystic ovary syndrome. *Fertility and Sterility*.

[11] Pasquali R, Gambineri A (2006). Polycystic ovary syndrome: a multifaceted disease from adolescence to adult age. *Annals of the New York Academy of Sciences*.

[12] Abir R, Ao A, Jin S (2005). Leptin and its receptors in human fetal and adult ovaries. *Fertility and Sterility*.

[13] Ram MR, Sundararaman PG, Malathi R (2005). Body fat distribution and leptin correlation in women with polycystic ovary syndrome: endocrine and biochemical evaluation in south Indian population. *Reproductive Medicine and Biology*.

[14] Li M-G, Ding G-L, Chen X-J (2007). Association of serum and follicular fluid leptin concentrations with granulosa cell phosphorylated signal transducer and activator of transcription 3 expression in fertile patients with polycystic ovarian syndrome. *Journal of Clinical Endocrinology and Metabolism*.

[15] De Placido G, Alviggi C, Clarizia R (2006). Intra-follicular leptin concentration as a predictive factor for in vitro oocyte fertilization in assisted reproductive techniques. *Journal of Endocrinological Investigation*.

[16] Savastano S, Valentino R, Di Somma C (2011). Serum 25-Hydroxyvitamin D Levels, phosphoprotein enriched in diabetes gene product (PED/PEA-15) and leptin-to-adiponectin ratio in women with PCOS. *Nutrition and Metabolism*.

[17] Lecke SB, Mattei F, Morsch DM, Spritzer PM (2011). Abdominal subcutaneous fat gene expression and circulating levels of leptin and adiponectin in polycystic ovary syndrome. *Fertility and Sterility*.

[18] Reverchon M, Cornuau M, Cloix L (2013). Visfatin is expressed in human granulosa cells: regulation by metformin through AMPK/SIRT1 pathways and its role in steroidogenesis. *Molecular Human Reproduction*.

[19] Choi K-H, Joo B-S, Sun S-T (2012). Administration of visfatin during superovulation improves developmental competency of oocytes and fertility potential in aged female mice. *Fertility and Sterility*.

[20] Garruti G, Depalo R, Vita MG (2009). Adipose tissue, metabolic syndrome and polycystic ovary syndrome: from pathophysiology to treatment. *Reproductive BioMedicine Online*.

[21] Depalo R, Lorusso F, Palmisano M (2009). Follicular growth and oocyte maturation in GnRH agonist and antagonist protocols for invitro fertilisation and embryo transfer. *Gynecological Endocrinology*.

[22] The Expert Committee on the Diagnosis and Classification of Diabetes Mellitus (1997). Report of the expert committee on the diagnosis and classification of diabetes mellitus. *Diabetes Care*.

[23] Falagario D, Brucculeri AM, Depalo R, Trerotoli P, Cittadini E, Ruvolo G (2012). Sperm head vacuolization affects clinical outcome in ICSI cycle. A proposal of a cut-off value.

[24] Veeck LL (1986). *Atlas of the Human Oocyte and Early Conceptus*.

[25] Matthews DR, Hosker JP, Rudenski AS (1985). Homeostasis model assessment: insulin resistance and *β*-cell function from fasting plasma glucose and insulin concentrations in man. *Diabetologia*.

[26] Matsuda M, DeFronzo RA (1999). Insulin sensitivity indices obtained from oral glucose tolerance testing: comparison with the euglycemic insulin clamp. *Diabetes Care*.

[27] Armitage P, Berry G (1994). *Statistical Methods in Medical Research*.

[28] Dawson B, Trapp RG (2001). *Basic & Clinical Biostatistics*.

[29] Azziz R, Woods KS, Reyna R, Key TJ, Knochenhauer ES, Yildiz BO (2004). The prevalence and features of the polycystic ovary syndrome in an unselected population. *Journal of Clinical Endocrinology and Metabolism*.

[30] Wild RA, Carmina E, Diamanti-Kandarakis E (2010). Assessment of cardiovascular risk and prevention of cardiovascular disease in women with the polycystic ovary syndrome: a consensus statement by the androgen excess and polycystic ovary syndrome (AE-PCOS) society. *Journal of Clinical Endocrinology and Metabolism*.

[31] Alexander CJ, Tangchitnob EP, Lepor NE (2009). Polycystic ovary syndrome: a major unrecognized cardiovascular risk factor in women. *Reviews in Obstetrics & Gynecology*.

[32] Zimmet P, Alberti G, Shaw JA (2005). A new IDF worldwide definition of the metabolic syndrome: the rationale and the results. *Diabetes Voice*.

[33] Lin Q, Poon SL, Chen J, Cheng L, HoYuen B, Leung PCK (2009). Leptin interferes with 3′,5′-Cyclic Adenosine Monophosphate (cAMP) signaling to inhibit steroidogenesis in human granulosa cells. *Reproductive Biology and Endocrinology*.

[34] Cervero A, Dominguez F, Horcajadas JA, Quinonero A, Pellicer A, Simon C (2006). The role of the leptin in reproduction. *Current Opinion in Obstetrics and Gynecology*.

[35] Sam S, Dunaif A (2003). Polycystic ovary syndrome: syndrome XX?. *Trends in Endocrinology and Metabolism*.

[36] Corbould A, Dunaif A (2007). The adipose cell lineage is not intrinsically insulin resistant in polycystic ovary syndrome. *Metabolism: Clinical and Experimental*.

